# Scheme of the Antiseptic Method of Womb Treatment

**Published:** 1890-11

**Authors:** 


Scheme of the Antiseptic Method of Wound Treatment. By Dr. Albert Hoffa, Private Docent of Surgery in the University Würzburg. Translated from the German, with Additions, by Special Permission of the Author, by Aug. Schachner, M. D., Ph. G., Louisville, Ky. The Bradley & Gilbert Co., Louisville, Ky.

The Scheme is a concise detail of aseptic and antiseptic materials and methods of operating arranged in the form of a chart.

The various antiseptic powders, solutions, salves, etc., their ormulæ and methods of preparation are given in tabulated orm; as are also the preparation of aseptic and antiseptic igature, suture and drainage materials and dressings. Also Lister's dressings and the different modifications thereof, etc., etc.

The Scheme will immediately commend itself to every practical surgeon, and must prove invaluable to the beginner who wishes to perfect himself in the art of aseptic and antiseptic surgery. F. W. M.

---

### FATAL AFTER-EFFECT OF CHLOROFORM.

C. Thiem and G. Fisher have reported a case of death four days after chloroform narcosis. They made various experiments and found, in agreement with previous investigators, that fatty degeneratiŏn of the liver, kidneys and glands of the stomach and small intestines occurred. They conclude that frequent chloroforming must be avoided, a patient being ready for another chloforming only when the urine no longer "reduces" Fehling's solution (sugar). Any state of depression renders great caution necessary in chloroforming.

---

### VARICOSE VEINS.

Angelo Nannoti has reported eight cases of depression in the tibia, caused by varicose veins, the veins lying in grooves. Has found the treatment of these veins by injection of equal parts of water and chloral-hydrate good, but he preferred Schede's method of extirpation.

---

### ALEXANDER'S OPERATION.

Richlot mentions two operations of this kind to relieve retroflexion of the uterus. First failed because of fatty degeneration of the round ligament. Second seemed successful at first, but the uterus soon resumed former position; it contained a small myoma. Thinks these operations do not speak in favor of the method.

